# Image Analysis of Pellet Size for a Control System in Industrial Feed Production

**DOI:** 10.1371/journal.pone.0026492

**Published:** 2011-10-21

**Authors:** Martin Georg Ljungqvist, Michael Engelbrecht Nielsen, Bjarne Kjær Ersbøll, Stina Frosch

**Affiliations:** 1 Department of Informatics and Mathematical Modelling, Technical University of Denmark (DTU), Kongens Lyngby, Denmark; 2 Division of Industrial Food Research, National Food Institute, Technical University of Denmark (DTU), Kongens Lyngby, Denmark; University of Bristol, United Kingdom

## Abstract

When producing aquaculture fish feed pellets, the size of the output product is of immense importance. As the production method cannot produce pellets of constant and uniform size using constant machine settings, there is a demand for size control. Fish fed with feed pellets of improper size are prone to not grow as expected, which is undesirable to the aquaculture industry. In this paper an image analysis method is proposed for automatic size-monitoring of pellets. This is called granulometry and the method used here is based on the mathematical morphological opening operation. In the proposed method, no image object segmentation is needed. The results show that it is possible to extract a general size distribution from an image of piled disordered pellets representing both length and diameter of the pellets in combination as an area.

## Introduction

In the aquaculture industry it is of outmost importance that the fish get feed of proper size. The feed is usually in pellet form, where the pellets contain the nutrients that the fish need to grow and stay healthy. The size of the pellets is adapted to the size of the fish so that the fish can grow as expected. It has been shown that growth rate of fish is closely related to the pellet size of feed [1]–[3]. Therefore, when producing feed pellets for aquaculture there is a need to control the size of the output product, and this is a challenging task.

An extruder machine is commonly used for fish feed production. The feed material is extruded through a die plate with holes of a certain diameter which determines the diameter of the pellets. On the other side of the disk is a set of rotating knives that cut the material into shorter cylinder-shaped pellets. The length of the pellets is affected both by the velocity of the knives and the pressure inside the machine.

When the extruder machine has been running for some time, the holes in the die plate get clogged with raw material. The time-frame for this to happen depends on the composition of the raw material and the pellet size produced. This clogging of holes restrains further material – either completely or partly – from flowing through the holes and therefore affects the output of the machine.

Additionally, the pressure inside the extruder rises during operation, inducing a rise in the velocity of the feed as it passes through the holes, resulting in a drift of the pellet size. Moreover, the temperature of the machine increases, and this might affect its physical properties, both velocity and pressure. Changes in pressure and temperature result in the problem that the pellet size changes over time during a batch production.

Today, size monitoring is done by manual inspection in order to adjust the settings or restart the machine. This is both labour-demanding and relies on experienced assessors. An automatic vision system for on-line quality control would be of great benefit to the industry, both for process control and product optimisation. If automatic size measurement could indicate when the pellet size is outside the defined range, this information could be used to adjust machine settings such as the knife speed, screw speed, filling rate or other means of controlling the pellet expansion process, thereby controlling the pellet size and ensuring uniformity.

Measuring the size distribution of small particles is often referred to as granulometry or sometimes as particle size distribution analysis [4]. The proposed method is based on image analysis using mathematical morphology and in particular using the so-called morphological openings used for size distribution analysis. This technique was proposed by Matheron (1975) [5], a vast amount of work on granulometry on binary images was also done by Serra (1982) [6] and the technique has been further developed for grey-scale images [7], [8].

Morphological openings are widely used for granulometry in image analysis and have been used for many applications [9]–[13]. These all use an image segmentation method before performing the morphological opening.

Another approach to granulometry is the use of frequency transform analysis, as can be seen in Zadoro
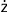
ny et al. (2002) [14], where a specific segmentation method is not needed. The same paper also used the technique of scale-space [15]–[17], which can likewise be seen in Clemmensen et al. (2009) [18]. A similar approach without segmentation can be seen in Jägersand (1995) [19].

Measuring fish feed pellet size by image analysis has previously been done by Parsonage (2001), who used a stepwise grey-scale threshold to segment the pellets in underwater images [20]. Moreover it has also been done in the work by Foster et al. (1995) using a grey-scale threshold and classification based on binary shape and size of image objects [21]. The threshold method, with corresponding binary shape features, is limited since it only works on single pellets and is therefore not suitable for the problem presented in this paper; measuring piled, disordered pellets.

Besides these, the area of determining the size of fish feed pellets has, to the authors’ knowledge, not been further investigated. Image analysis has not previously been used in production quality control of feed pellets.

The aim of the present work is to develop an on-line control system based on image analysis for real-time inspection of pellet size during industrial production. This will be done using the well-known method of morphological opening, which can be interpreted as a mathematical analogy to sieving. By calibrating the method to a threshold of what is and what is not an acceptable size, the industry can use the method to get automatic and instant feedback about pellet size in order to adjust the machine settings during production.

Moreover, since the method is to be on-line in the production plant where the pellets are still moist, it should be investigated how the size measurements of moist and dry pellets correlate. It is of interest to investigate if it is possible to predict the size of pellets in dry condition from measurements made of moist pellets.

## Materials and Methods

The shape of the pellets is close to cylindrical with a length and a diameter, where the diameter is the product size referred to in the industry. It is assumed that both length and diameter change during the production time.

Initially, the size increase of pellets during production was investigated both by image analysis and by manual measurement. The image analysis was done using grey-scale images of 24 pellets of a certain size category and measuring the length and width of pellets in pixels using a threshold and binary operation for the major and minor axes of the pellets. The size of pellets at production start was compared to the size at production stop of the same batch. Manual measurement was done on 51 pellets from each time sample of batch C and D using a Mahr 16EX calliper (Mahr GmbH Esslingen) with a resolution of 0.01 mm and error limit of 0.02 mm.

### Image Acquisition

All images were taken using a Point Grey Scorpion SCOR-20SOM grey-scale camera, with the pellets placed in a plastic petri dish (diameter 9 cm) inside an integrated sphere (Ulbricht sphere) with uniform diffuse lighting from light-emitting diodes placed around the sphere. This kept the amount of shadow to a minimum, and the upper part of the pellet pile were almost without shadows. The combined camera and light set-up used was the VideometerLab device (Videometer A/S, Hørsholm, Denmark). The image dimension used was 800×600 pixels. In this situation one pixel equals approximately 0.072×0.072 millimetres, hence the image represents approximately a 57.6×43.2 mm area.

### Sample Preparation

Pellets were collected from a production batch at specific time intervals and a total of four batches were analysed, see Table 1. Images of dry pellets were taken from all four batches. Additionally, for batches C and D images were captured directly after the pellets were extruded, meaning that the pellets had a high level of moisture, whereas the finished product is dry.

**Table 1:** The batches analysed.

	Size	Sampling time	Observations	Repetitions	Total time
	(mm)	(min)			(min)
Batch A	1.1	30	10	5	270
Batch B	1.1	60	6	1	300
Batch C	1.1	5	11	1	50
Batch D	3.0	1–5	27	1	45

Product size (millimetres), time between sampling (minutes), number of observations, number of repetitions for each observation and total batch time (minutes).

Two different pellet product sizes were used to investigate whether there would be any difference related to product size and the result of the proposed method. The product size refers to the nominal diameter, which was 1.1 mm and 3.0 mm, respectively, for the analysed pellet batches.

### Image Analysis

By analysing images of pellets, with sizes ranging from normal to large, a model for distinguishing the relative size of pellets in an image can be made.

To start with, the images were adjusted for contrast before being analysed using contrast-limited adaptive histogram equalization (CLAHE).

The granulometry method used was a morphological opening operation. Since we did not know the orientation of the piled pellets in an image, this operation was performed so that it was independent of rotation.

**Figure 1:**
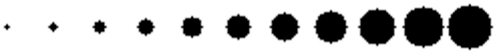
Structuring element. Disk of radius 1–11 pixels created using the Euclidean distance.

**Figure 2:**
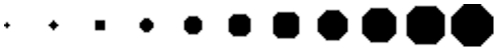
Structuring element. Disk of radius 1–11 pixels created using a decomposition method with a neighbourhood of 4 pixels.

The morphological opening operation performs an erosion operation followed by a dilation operation using a so-called structuring element. A structuring element is a mathematical binary shape; here we used the shape of a disk, see Figures 1 and 2. If the structuring element can fit inside an area of the image then the whole structural element will appear in the result of the transformation.

The parts of the original image that the operator does not fit inside will not be a part of the result. This means, that if the objects in the image are smaller than the element size, then the result will be of low intensity. This is achieved because the erosion operation leaves a disk-shaped area consisting of the lowest pixel value found inside the disk-element region of the image for each viable position of the element inside the image. Dilation does the same thing but uses the maximum value for each region.

The erosion, dilation and opening for an image 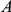 and a structuring element 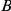 are defined as:
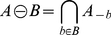
(1)

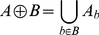
(2)

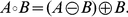
(3)


**Figure 3:**
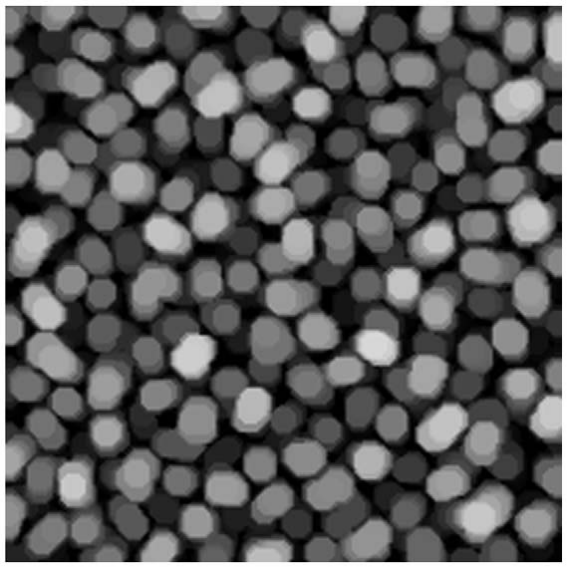
Image opening. Morphological opening using a disk with radius of 5 pixels on start-up sample pellets.

**Figure 4:**
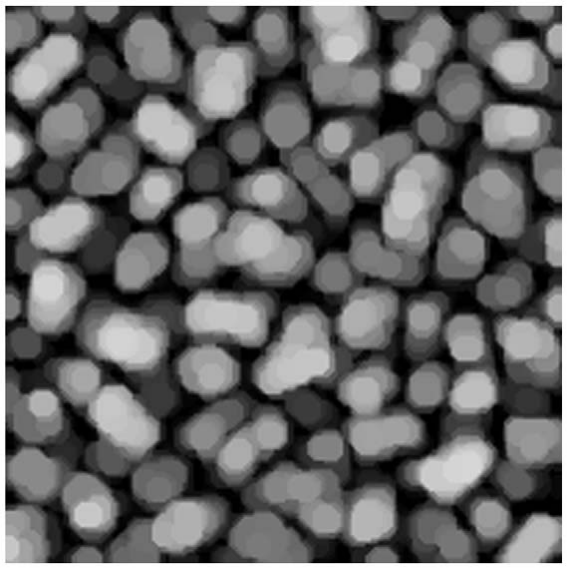
Image opening. Morphological opening using a disk with radius of 5 pixels on stopping sample pellets.

**Figure 5:**
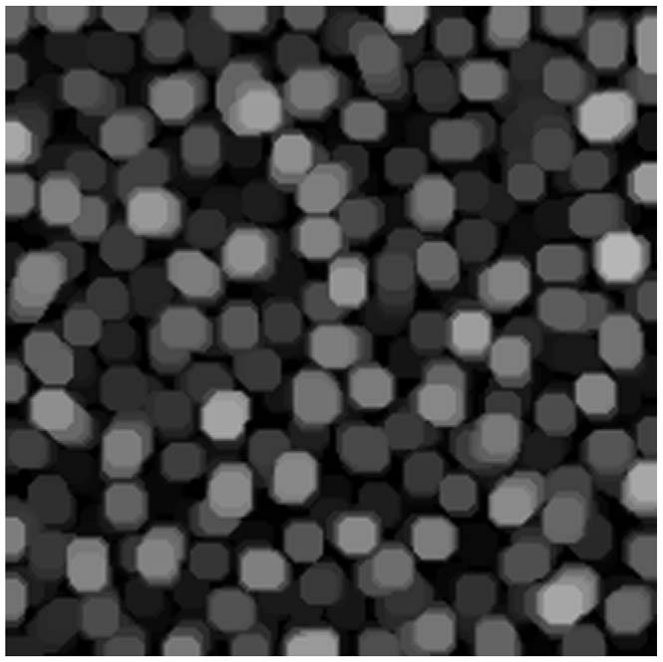
Image opening. Morphological opening using a disk with radius of 6 pixels on start-up sample pellets.

**Figure 6:**
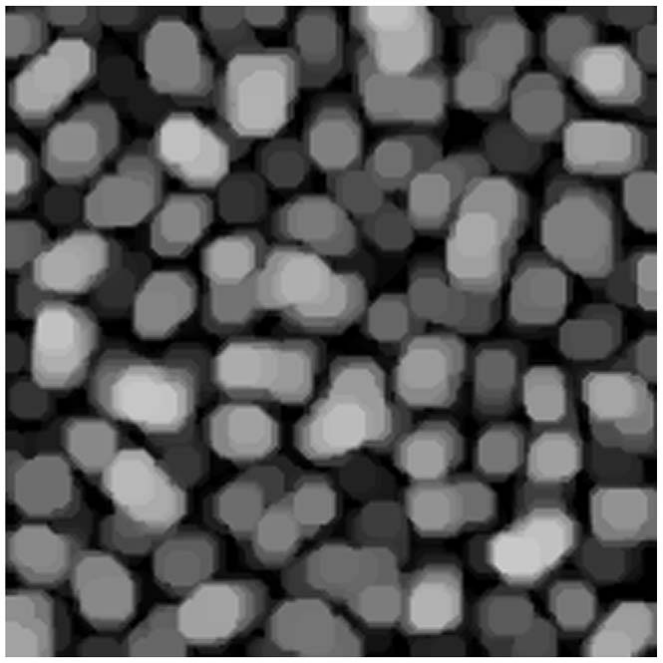
Image opening. Morphological opening using a disk with radius of 6 pixels on stopping sample pellets.

The opening operation can be interpreted as finding objects of a certain size and, to some extent, of a certain shape. See Figures 3–6 for examples of resulting images of this method.

For comparison, different structuring elements were tested: disk, octagon, ball and diamond. All showed similar performance (results not shown), and therefore the disk operator was chosen, since it constitutes a good approximation of general pellet size with both increasing length and width.

By increasing the radius of the disk and summing the image intensity for each radius, an opening intensity curve is achieved. The advantage of this method is that it can be used on an image filled with pellets; segmentation is not necessary. The method will not give a size distribution based on each single pellet. Rather is it an overall assessment on a pile of pellets which makes a size index measure.

The opening intensity corresponding to each disk element size used is similar to what one would get using the scale-space method and represents a measure of the size information in the image. When a disk size cannot fit into most of the pellets in an image, the intensity output from the opening operation will be low. When the pellet size increases the location of the major intensity drop in the intensity curve changes. The opening intensity curve is also known as the granulometric curve. In order to analyse the changes in the decline of the intensity curve, we calculate the derivative of the opening intensity by calculating the differences between each opening operation.

Successive sample points over time have different opening intensity curves and this reflects the change in size distribution. Various methods for analysing the opening intensity curves have been tested and are described below.

Initially a structuring element disk with no approximation was used. The disk was created using the Euclidean distance, see Figure 1. Further on, a disk with some approximation was used to reduce calculation time, see Figure 2. The disk with approximation is less than a perfect disk but instead it is decomposable [22], which vastly increases the computational speed. The decomposition technique of Jones et al. (1996) was used [23].

### Pattern Spectrum

In granulometry, the derivative is often replaced by the so-called Pattern Spectrum (PS) [24]. The pattern spectrum is defined as the difference between the intensity values of successive morphological opening operations divided by the sum of all pixel values in the original image. The pattern spectrum is equivalent to the negative derivative of the opening intensity normalised by the total pixel sum. The pattern spectrum is also known as Pecstrum and is interpreted as the estimated particle size distribution function.

The discrete pattern spectrum of image 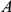 using structuring element 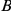 is defined as:
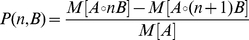
(4)where 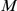 represents the area measured in the intermediate operations, and 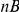 is the structuring element with the size increased 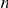 times.

When analysing pellets of increasing size, we can use that the maxima in the pattern spectrum changes location. For analysing the change in size over time, we use the opening intensity at the position of the pattern spectrum maximum as a size index, here called APM.

### Size Index

Another way of analysing the morphological opening intensity curves is to take the average of each of these curves to reveal the progression. Typically this would result in an increasing sequence.

In statistical process control, the median is often used instead of the mean to get more robust results. By taking the median value of each opening intensity curve, this would give a good measure of the size changes. This measure represents looking in the vertical direction at the middle of the successive intensity curves. Moreover, the Interquartile Range (IQR) can be used for analysing variation of the opening results. The IQR is constituted as the difference between the first and third quartiles of the curve [25].

**Figure 7:**
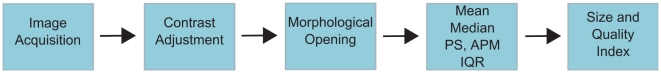
Method overview. Block diagram of the method used. The size index is calculated using one of the methods mean, median or APM. The pattern spectrum is used as a size distribution estimate. IQR is calculated to get a measurement of the spread of the size distribution and as another quality measure.

An overview of the proposed size index method can be seen in Figure 7.

All image analyses and statistics were carried out using Matlab 7.9 (The Mathworks Inc., Natick, MA, USA).

## Results

**Figure 8:**
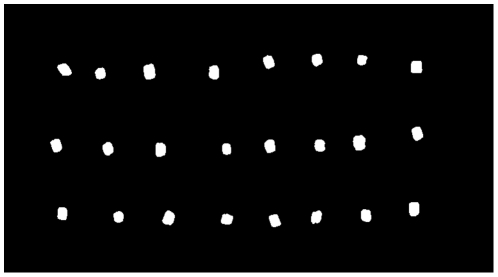
Pellets. Binary image of pellets at production start.

**Figure 9:**
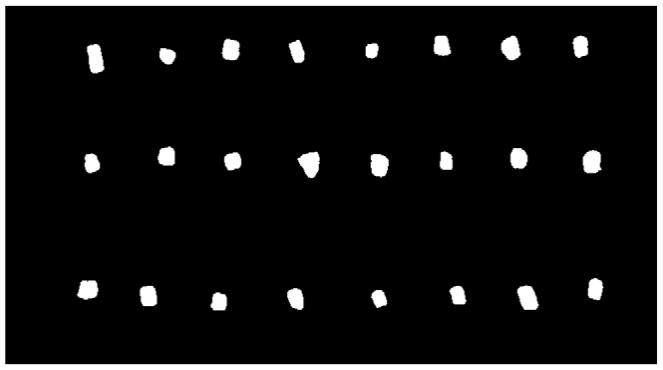
Pellets. Binary image of pellets at production stop.

In the initial image analysis of single pellets, the size of 24 pellets at production start was compared to the size of 24 pellets at production stop, see Figures 8 and 9. The comparison using image analysis shows an increase in both length and width (diameter). Both length and width (diameter) were significantly different at a 0.1% significance level, indicating that the area of a pellet in an image increases with time.

### Calliper Measurement

Manual measurements was done on the pellet width (diameter) and length using a calliper. The results show a clear increase in both the diameter and the area (length×width) of the pellets over time, see Table 2. The mean values of the calliper measurements at start and stop of both batch C and D in dry condition are significantly different at a 0.1% significance level. The statistical power for these calliper measurements of the diameter is set to 80%, which results in an estimated accuracy of 0.09 mm for batch C (1.1 mm) and 0.055 mm for batch D (3.0 mm) at a 5% significance level. This means, that we trust the calliper measurements as a base for comparison of the image analysis results.

**Table 2:** Image analysis and calliper measurements.

	Production	Calliper	Opening	Opening
	diam. (mm)	diam. (mm)	radius (pixels)	diam. (mm)
		Start	Start	Start
Batch A	1.1	-	7	1.008
Batch B	1.1	-	4	0.576
Batch C	1.1	1.236	7	1.008
Batch D	3.0	3.094	21	3.024
		Stop	Stop	Stop
Batch A	1.1	-	9	1.296
Batch B	1.1	-	6	0.864
Batch C	1.1	1.805	8	1.152
Batch D	3.0	3.610	25	3.600

Pellet size results in pixels and millimetres for start and stop of the batch production. Production diameter (mm) is the target size of the produced pellets. Calliper diameter (mm) is the manually measured size of 51 pellets. Morphological opening radius (pixels) is the radius with the highest density in the pattern spectrum. Opening diameter (mm) is the opening radius converted from pixels to diameter in millimetres. Calliper measurements were only done on batch C and D. Pellets in dry condition.

### Opening Intensity Curve

**Figure 10:**
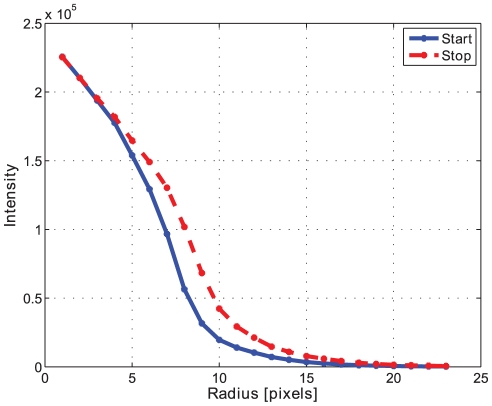
Opening intensity. Morphological opening intensity for the start-up sample 1 (blue) and the stopping sample 10 (red) in Batch A.

**Figure 11:**
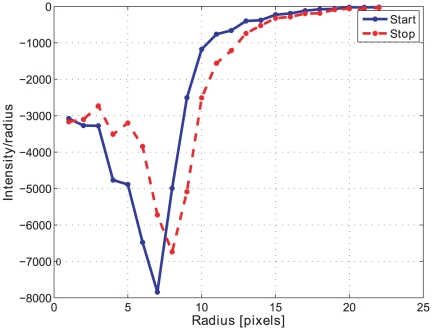
Opening derivative. The first derivative of the start sample 1 (blue) and stop sample 10 (red) opening intensity in Figure 10.

**Figure 12:**
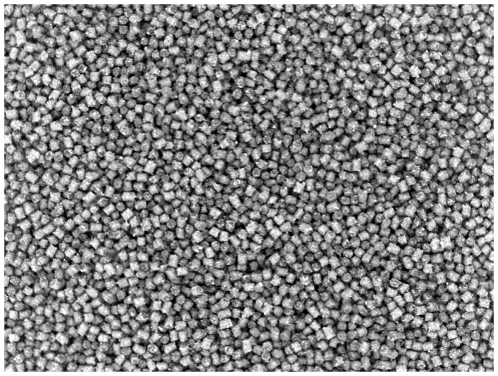
Pellets. Image of pellets at production start. Batch A.

**Figure 13:**
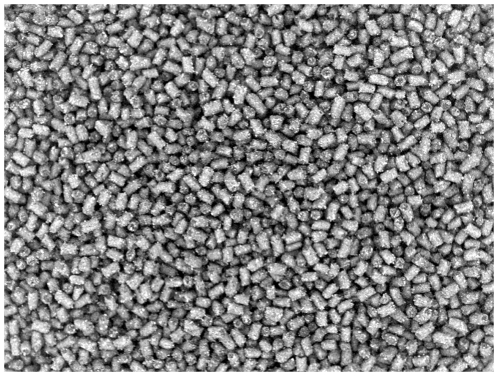
Pellets. Image of pellets at production stop. Batch A.

The morphological openings were applied to all batches in order to calculate the size index. Comparing the morphological opening curves for start and stop samples reflects a change in the pellet size distribution, see Figure 10 for batch A. The derivative of the opening intensity of batch A can be seen in Figure 11. The corresponding images for start and stop samples of batch A can be seen in Figures 12 and 13.

**Figure 14:**
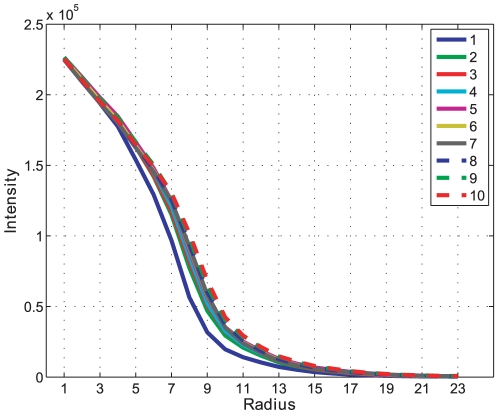
Opening intensity. Morphological opening intensity for all time samples (1–10) in Batch A. This is the result of the image opening operation.

**Figure 15:**
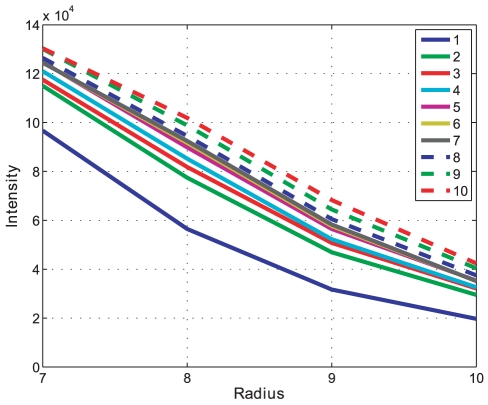
Opening intensity. Close-up of Figure 14 showing the changes in opening intensity for all time samples. Morphological opening intensity for all time samples (1–10) in Batch A.

**Figure 16:**
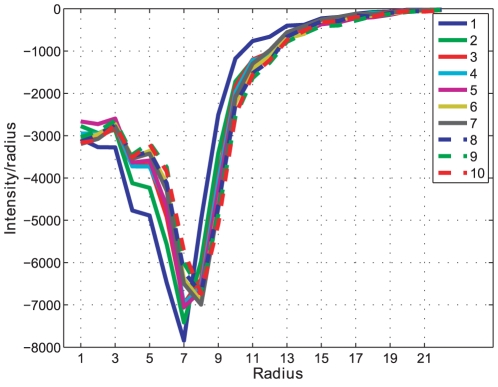
Opening derivative. The first derivative of the opening intensity for all time samples (1–10) in Batch A.

The successive morphological opening intensity curves are an indication of pellet size changes in an image and the result for batch A can be seen in Figure 14. A close-up on the morphological opening intensity for batch A showing the size changes over time can be seen in Figure 15. Each derivative extremum in the intensity derivative indicates a change in the opening intensity, the result for batch A can be seen in Figure 16.

**Figure 17:**
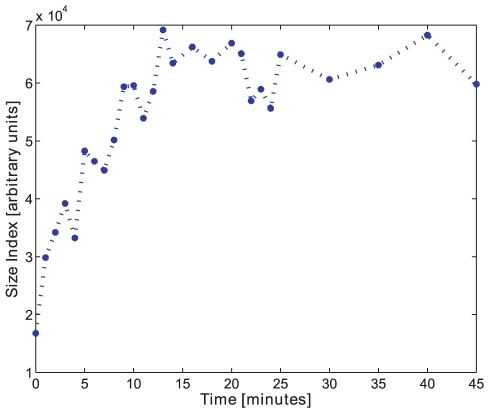
Size index. The size index using the median of morphological opening intensity on pellets from batch D.

The size index calculated by the opening intensity mean, as well as the size index calculated by the opening intensity median, both indicate the trend of the size change in all tests, see Figure 17 for batch D.

**Figure 18:**
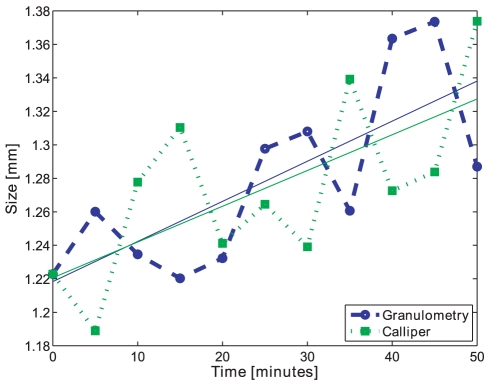
Size index comparison. The median of morphological opening intensity of dry pellets (blue) compared to the calliper diameter measurements of dry pellets (green), and their trend-lines, batch C. Size index values normalised for comparison purpose.

The opening intensity mean on dry pellets for batches C and D was compared to the calliper measurements’ mean area value. Since the opening operation measures both diameter and length of the pellets in an image, both are included as area in this correlation. The correlation of the two curves of batch C is 0.52, see Figure 18, and for Batch D the correlation is also 0.52. Moreover it can be seen that the trend-lines of the image analysis result correlate positively with the trend-lines of the calliper measurements’ mean value. For both batches C and D, the trend-lines of the image analysis and calliper measurements are increasing. An overview of the comparison between image analysis and calliper measurements can be seen in Table 2. For full results on correlation between image analysis and calliper measurements, see Table 3.

**Table 3:** Image analysis and calliper measurements correlation.

	Full	Decomposable	Trend-line
	Disk	Disk	
Batch C			
Mean	0.5185	0.5199	1.0000
Median	0.5597	0.5610	1.0000
APM	0.5118	0.5102	1.0000
IQR	0.3886	0.3888	1.0000
Batch D			
Mean	0.5172	0.5129	1.0000
Median	0.4381	0.4369	1.0000
APM	0.4821	0.4757	1.0000
IQR	0.4428	0.4410	1.0000

Size change correlation between image analysis (size index and spread) and calliper area measurements (length × diameter). Correlation for different disk approximation types for batches C and D are shown. The size index is calculated using morphological openings and then using the mean, median or the opening intensity at the position of the pattern spectrum maximum (APM). The Interquartile Range (IQR) is used as a variation measurement. Pellets in dry condition.

Comparing the size index with the calliper diameter measurements gives that the Root Mean Square Error (RMSE), i.e. the deviation between the two methods, is 0.07 mm for batch C with production size 1.1 mm, and 0.06 mm for batch D with production size 3.0 mm, see Table 4. This means that the image analysis method shows good estimated accuracy.

**Table 4:** Image analysis method compared to calliper measurements.

	SD (mm)	RMSE (mm)
Batch C (1.1 mm)		
Mean	0.0547	0.0749
Calliper	0.0528	-
Batch D (3.0 mm)		
Mean	0.1236	0.0590
Calliper	0.1390	-

The deviation between the size index using mean of the morphological opening intensity and the calliper diameter measurements. SD is the Standard Deviation and RMSE is the Root Mean Square Error. The size index was normalised using the mean of the calliper measurements in order to get a scale with millimetres. A disk without approximation has been used for the opening operation. Pellets in dry condition.

Both the mean and median of the morphological opening intensity show similar patterns, the correlation between the two is at least 0.92 for the batches analysed, see Table 5.

**Table 5:** Image analysis method correlation.

	Moist	Dry
	Pellets	Pellets
Batch A		
Median	-	0.9907
APM	-	0.9901
Batch B		
Median	-	0.9942
APM	-	0.9972
Batch C		
Median	0.9253	0.9494
APM	0.9864	0.9952
Batch D		
Median	0.9703	0.9493
APM	0.9743	0.9658

The correlation of various ways to calculate the size index. The correlation coefficient is here calculated between the mean of the morphological opening intensity and the median or APM of the same intensity. A disk without approximation was used.

**Figure 19:**
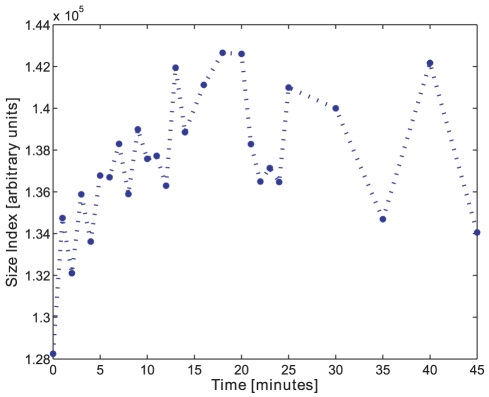
Size variation. The size variation using the Interquartile Range (IQR) on batch D.

The IQR of the opening intensity is a variation measure and shows that the pellet size variation increases over time in a similar manner as the size, see Figure 19.

### Pattern Spectrum

**Figure 20:**
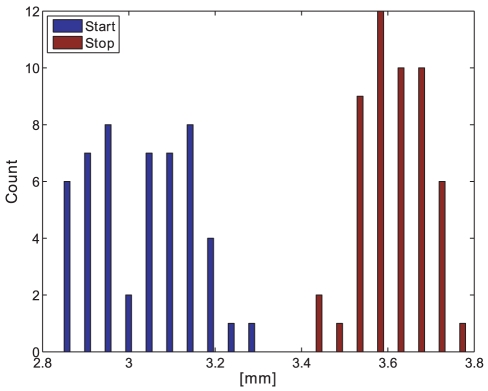
Calliper measurement. The manual, calliper-measured diameter of the start (blue) and stop samples (red) of 51 pellets from batch D. Pellets in dry condition.

**Figure 21:**
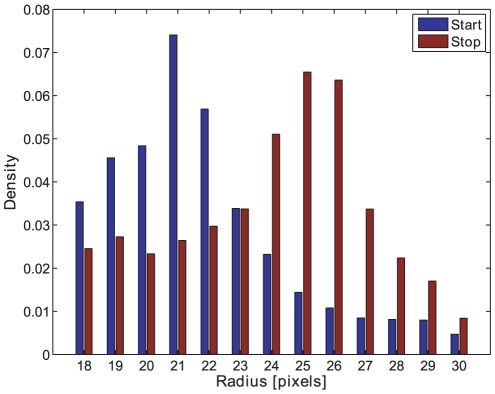
Pattern spectrum. The pattern spectrum showing the estimated area size distribution of start (blue) and stop (red) samples of batch D. Using image analysis with morphological opening. An image area of pellets of approximately 57.6×43.2 mm was used (800×600 pixels).

The pattern spectrum shows a good estimation of the size distribution. The pattern spectrum size distribution is similar to the one obtained with the calliper measurements, see Figure 20 and compare with the pattern spectrum in Figure 21. Analysing the values of the pattern spectrum to the right of the maximum value results in a good size change estimation similar to taking the mean or median of the morphological opening intensity curve. Also the opening intensity at the pattern spectrum maximum (APM) shows similar results, see Table 2. The trend-lines of APM and calliper measurements are both increasing.

### Dry and Moist Condition

**Figure 22:**
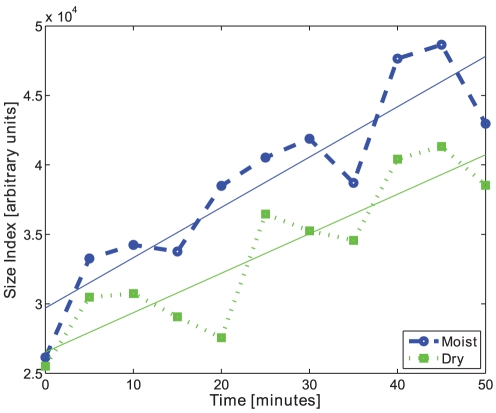
Size index of dry and moist. The median of the morphological opening intensity for batch C in moist (blue) and dry condition (green), and their trend-lines.

Batches C and D were analysed by the proposed method both in moist and dry conditions. The results obtained were not completely similar but the trends where almost identical, see Figure 22. The median of the opening operation intensity curves for moist and dry condition correlate at 0.96 for both batch C and batch D. It seems that the moist condition renders slightly higher size results than the dry condition for both batch C and batch D, however the difference is not large. This means that measurements of the moist pellets can be related to the dry pellets and the method can therefore be used for on-line quality control.

### Summing Up

It is concluded that the image opening intensity mean, intensity median and APM all perform well as a size index measure on a dish filled with pellets. Intensity median had the highest correlation with calliper measurements for batch C, and APM had the highest correlation for batch D, see Table 3. While APM performs similarly to intensity mean and intensity median, it needs slightly more computation without gaining any information in comparison. We also see that measurements on moist pellets have a high correlation with measurements on dry pellets and that we can therefore predict the dry pellet size from measurements on the moist pellets.

No particular difference could be seen in the behaviour of the opening measurements between product size 1.1 mm (batches A, B, C) and 3.0 mm (batch D). This implies, that the proposed method works for different production size categories.

Furthermore, based on the results obtained, it is concluded that the contrast level of the images affects the robustness of the size measure. The contrast level of the images is thus an important parameter for this method to perform well. For robust results of the size measurement, the image contrast should be as high as possible.

## Discussion

Previous work in this field has used segmentation before the morphological opening step, which could be a source of error and it also consumes computation time. In the present study it is demonstrated that a general size indication can be found using the morphological opening method on piled pellets without the segmentation procedure. Moreover it is shown that, of all the methods tested, the mean and median is the best method for analysing the opening intensity curve in order to estimate the pellet size progression.

When the pellets are piled, there is an angular distribution of how many are lying down, standing up, and at angles in between. It is therefore required that the model works even if the pellets are rotated. The measurement using the proposed method is of a projected area of arbitrarily rotated pellets. As the product size of the pellets changes, the proportions will change accordingly. This affects the distribution of lying and standing pellets and how they cast shadows on each other. If the diameter and/or the length have increased, the pellet volume will increase and thus the projected area in the image will increase. Therefore we measure the general size change on pellets randomly distributed in the image space.

The results clearly show that it is possible to extract a general size index from an image of piled pellets. The proposed method is suitable for on-line measurement of pellet size changes and will be of high importance to the pellet producing industry. This method is expected to work generally for granulometry situations and could be used for further applications.

The resulting size index can be used to calibrate a production batch for start-up size and a threshold size. The size measurement information can be used to adjust production parameters, as well as to make a quality characteristic control chart for size monitoring in the production. The size index seems to have a logarithmic trend for all the batches analysed. Further on it will be natural to use statistical quality control methods such as a moving average to analyse the size index in the production.

It was shown that the results obtained for moist and dry pellets are highly correlated. This result makes it possible to use the proposed method on moist pellets in the production and still be able to give a measurement of the size of the pellets in dry condition. The decrease in pellet size indication between moist and dry condition is interpreted such that the pellet size is smaller when water has evaporated from the pellet.

The difference in robustness of the results could be related to the total production time of the sampled batch, where samples from a batch with long production time would be more robust since short fluctuations in size would not be as influential here as in batches C and D with shorter time between samples.

The results of using an approximated (and decomposable) disk with a non-approximated disk are similar, see Table 3, however the pattern spectrum shows more artefacts when using an approximated disk. The approximation’s deviation from the theoretical disk area introduces a slight irregularity in the opening intensity curve and is hence clearly visible in its derivative with re-occurring spikes for certain radius values. However, these artefacts are at equal positions for all measurements and are assumed not to influence the result of the measuring method. A good indication of this can be seen in the disk comparison in Table 3, where it can be seen that the differences in correlation with the calliper measurements are very small between using a non-approximated disk and an approximated disk. These irregularities can be avoided, though, if non-integer radius values are used, i.e. the radii should be estimated from actual area of the decomposable disks.

The reason for using an approximated disk is to reduce computation time. The computational time using a non-approximated disk is about 10-times longer for batches of size 1.1 mm (A, B, C), and about 30-times longer for the 3.0 mm batch (D). The computational time for a morphological opening is dependent on the image size and the size of the structuring element. This means that the time penalty is worse when detecting large objects while using a non-approximated disk. Since the difference in the achieved size index between using an approximated disk and a non-approximated disk is small, the proposed method using an approximated disk shows acceptable calculation times for on-line measurement in the industry.

A more smooth behaviour of an opening intensity derivative can be achieved by performing a spline polynomial estimation of the intensity curve and then deriving this analytically. Polynomial estimation of morphological opening results can e.g. be seen in Morales-Hernández et al. (2010) [11].

The proposed method will not be affected if, due to moisture, pellets aggregate such that they could be interpreted as one large pellet. Such a cluster will not be interpreted as a large pellet, since the structure of the constituent pellets will still be visible.

Further investigation on how the following factors impact the result should be performed: lighting, camera, position of camera and pellets as well as pellet type. The lighting and hence the shadows is a topic believed to impact the result and should be investigated in more detail. Further investigations could e.g. be done using the scale-space method, frequency transforms or image profiles.
